# Metastatic lung tumor from hepatocellular carcinoma with tumor thrombus invasion in the pulmonary vein: a case report

**DOI:** 10.1186/s13019-023-02230-4

**Published:** 2023-04-28

**Authors:** Kazuto Ohtaka, Setsuyuki Ohtake, Yu Ishii, Saya Kaku, Yuta Takeuchi, Tomoko Mizota, Yoshiyuki Yamamura, Masaomi Ichinokawa, Tatsuya Yoshioka, Eiji Tamoto, Katsuhiko Murakawa, Koichi Ono

**Affiliations:** Department of Surgery, Obihiro Kosei General Hospital, West 14 South 10, Obihiro, Hokkaido 080-0024 Japan

**Keywords:** Hepatocellular carcinoma, Metastatic lung tumor, Tumor thrombus, Segmentectomy, Three-dimensional computed tomography

## Abstract

**Background:**

Metastatic lung tumor with a tumor thrombus in the peripheral pulmonary vein is very rare. We present a case of a metastatic lung tumor from hepatocellular carcinoma (HCC) with tumor thrombus invasion in the pulmonary vein that was diagnosed preoperatively and underwent complete resection by segmentectomy.

**Case presentation:**

A 77-year-old man underwent laparoscopic lateral segment hepatectomy for HCC eight years ago. Protein induced by vitamin K absence or antagonist-II remained elevated from two years ago. Contrast-enhanced chest computed-tomography (CT) showed a 27 mm nodule in the right apical segment (S1). He was pathologically diagnosed with a metastatic lung tumor from HCC via transbronchoscopic biopsy. We planned to perform right S1 segmentectomy. Before surgery, contrast-enhanced CT in the pulmonary vessels phase for three-dimensional reconstruction showed that the tumor extended into the adjusting peripheral pulmonary vein, and we diagnosed tumor thrombus invasion in V1a. The surgery was conducted under 3-port video-assisted thoracic surgery. First, V1 was ligated and cut. A1 and B1 were cut. The intersegmental plane was cut with mechanical staplers. Pathological examination revealed moderately-differentiated metastatic HCC with tumor thrombus invasions in many pulmonary veins, including V1a. No additional postoperative treatments were performed.

**Conclusions:**

As malignant tumors tend to develop a tumor thrombus in the primary tumor, it might be necessary to perform contrast-enhanced CT in the pulmonary vessel phase to check for a tumor thrombus before the operation for metastatic lung tumors.

**Supplementary Information:**

The online version contains supplementary material available at 10.1186/s13019-023-02230-4.

## Background

Recurrence after treatment for hepatocellular carcinoma (HCC) mostly occurs in the liver, and extrahepatic metastases are relatively rare, reported at 24% [1]. The most frequent extrahepatic metastasis is lung metastasis [2]. HCC has the tendency to infiltrate and form a tumor thrombus in the portal and hepatic veins; however, metastatic lung tumors from HCC with tumor thrombus invasion in the pulmonary vessels are rare, and there was only one case in past reports [3, 4]. We report a case of a metastatic lung tumor from HCC with tumor thrombus invasion in the pulmonary vein that was diagnosed preoperatively and underwent complete resection by segmentectomy.

## Case presentation

**Fig. 1:**
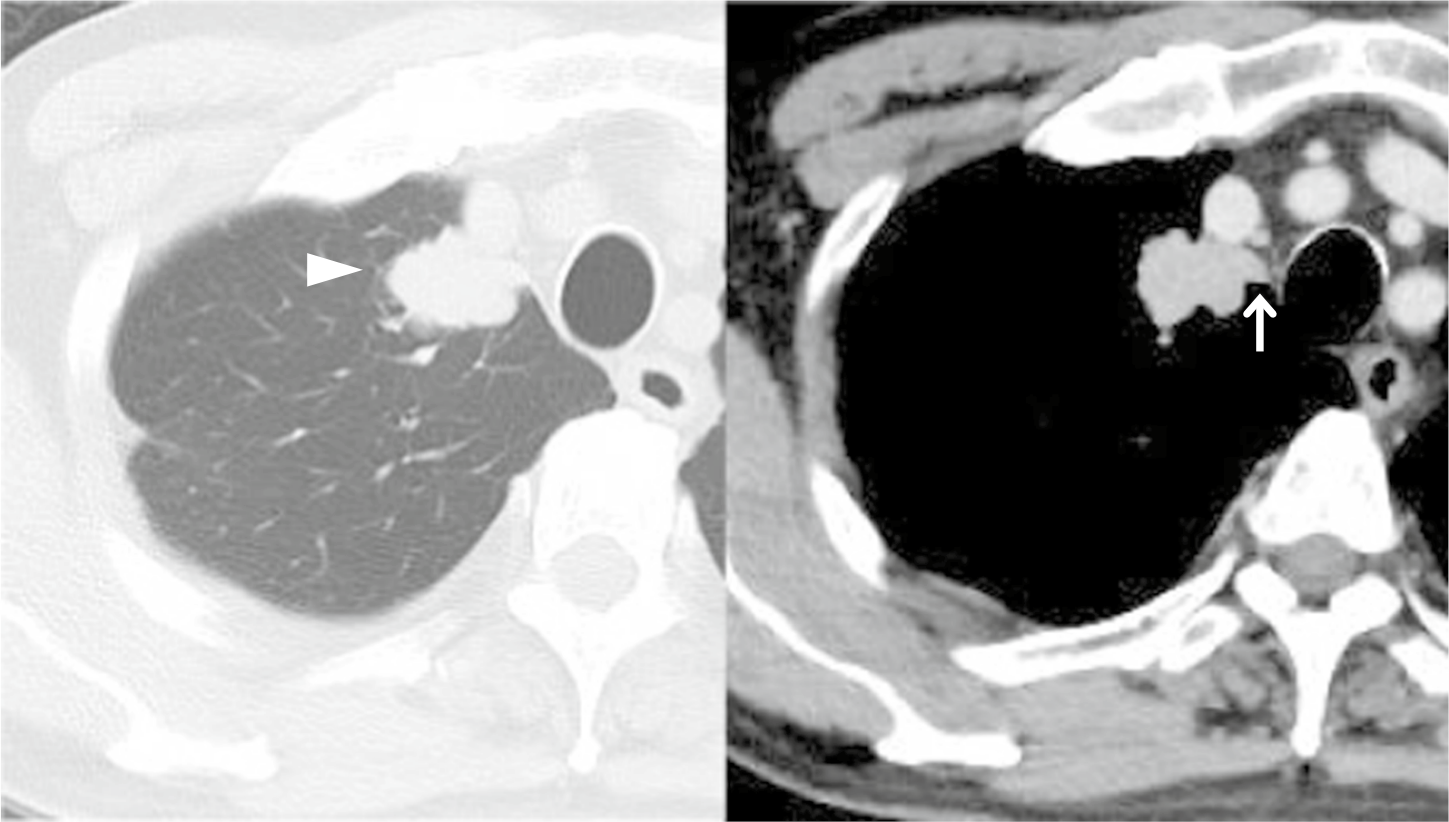
Contrast-enhanced chest CT. Contrast-enhanced chest computed tomography (CT) showing a 27 mm nodule (white arrowhead) with mediastinal pleural invasion (white arrow) in the right apical segment

**Fig. 2:**
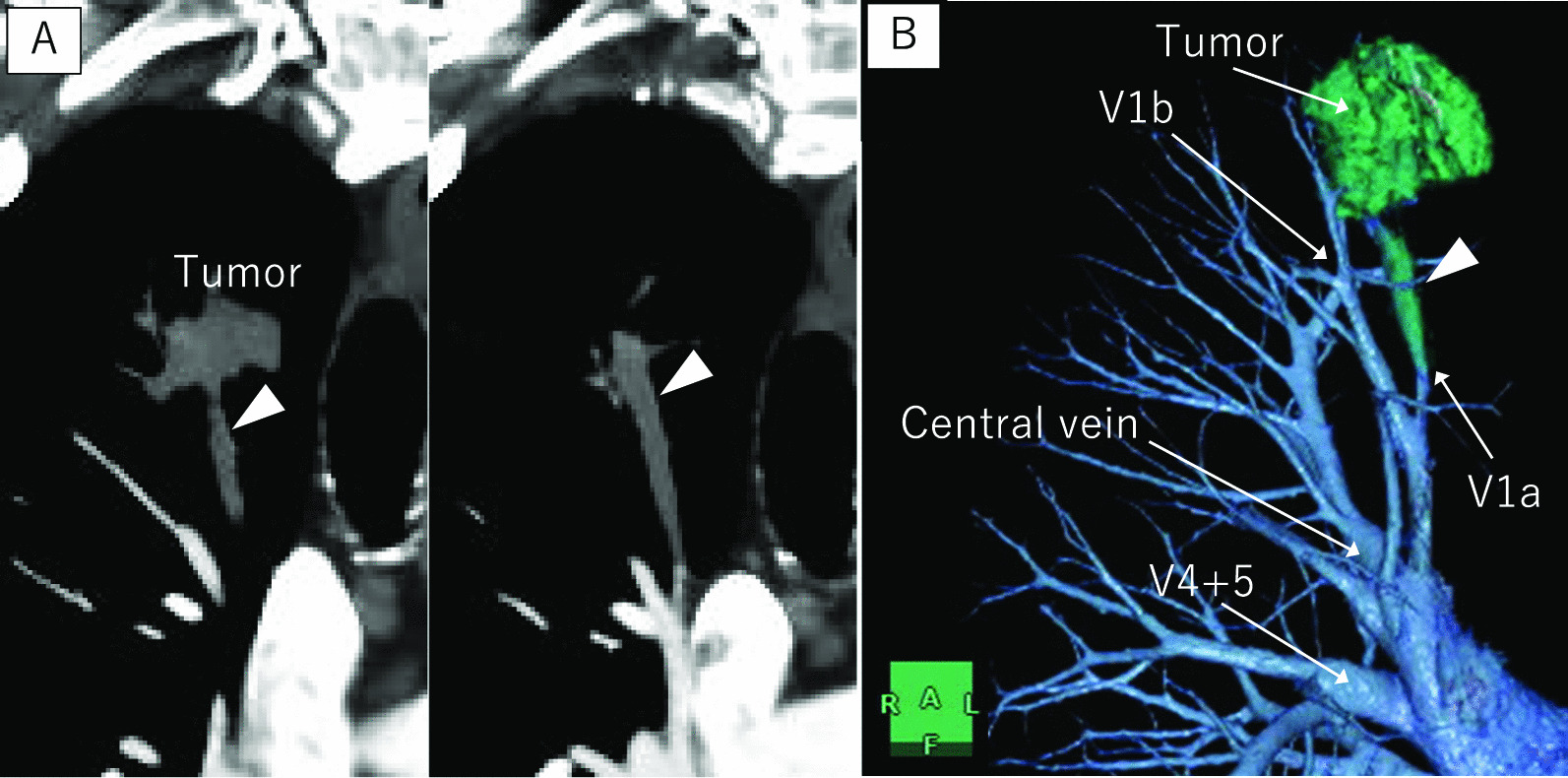
Contrast-enhanced CT in the pulmonary vessels phase for 3D reconstruction. **A** Coronal view of contrast-enhanced computed tomography (CT) in the pulmonary vessel phase showing the tumor extended into the adjacent peripheral pulmonary vein (white arrowhead); **B** Three-dimensional (3D) reconstruction of the pulmonary vein showing tumor thrombus invasion in V1a (white arrowhead)

**Fig. 3:**
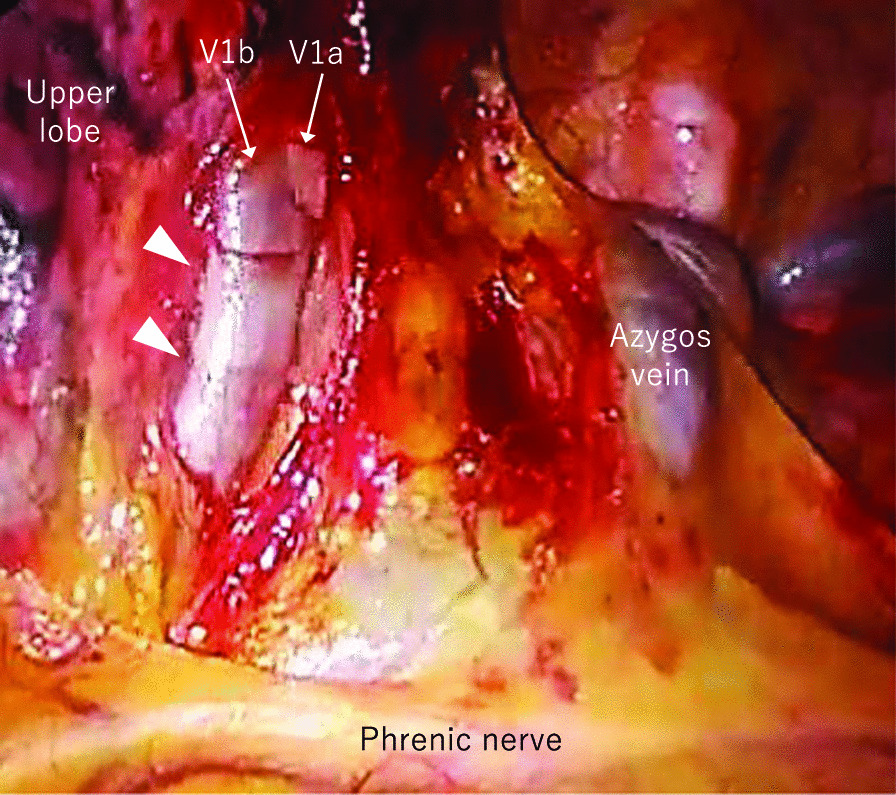
Intraoperative finding after exposure of V1. V1 looks slightly-dilated (white arrowhead). Tumor thrombus in V1 is not recognized

**Fig. 4:**
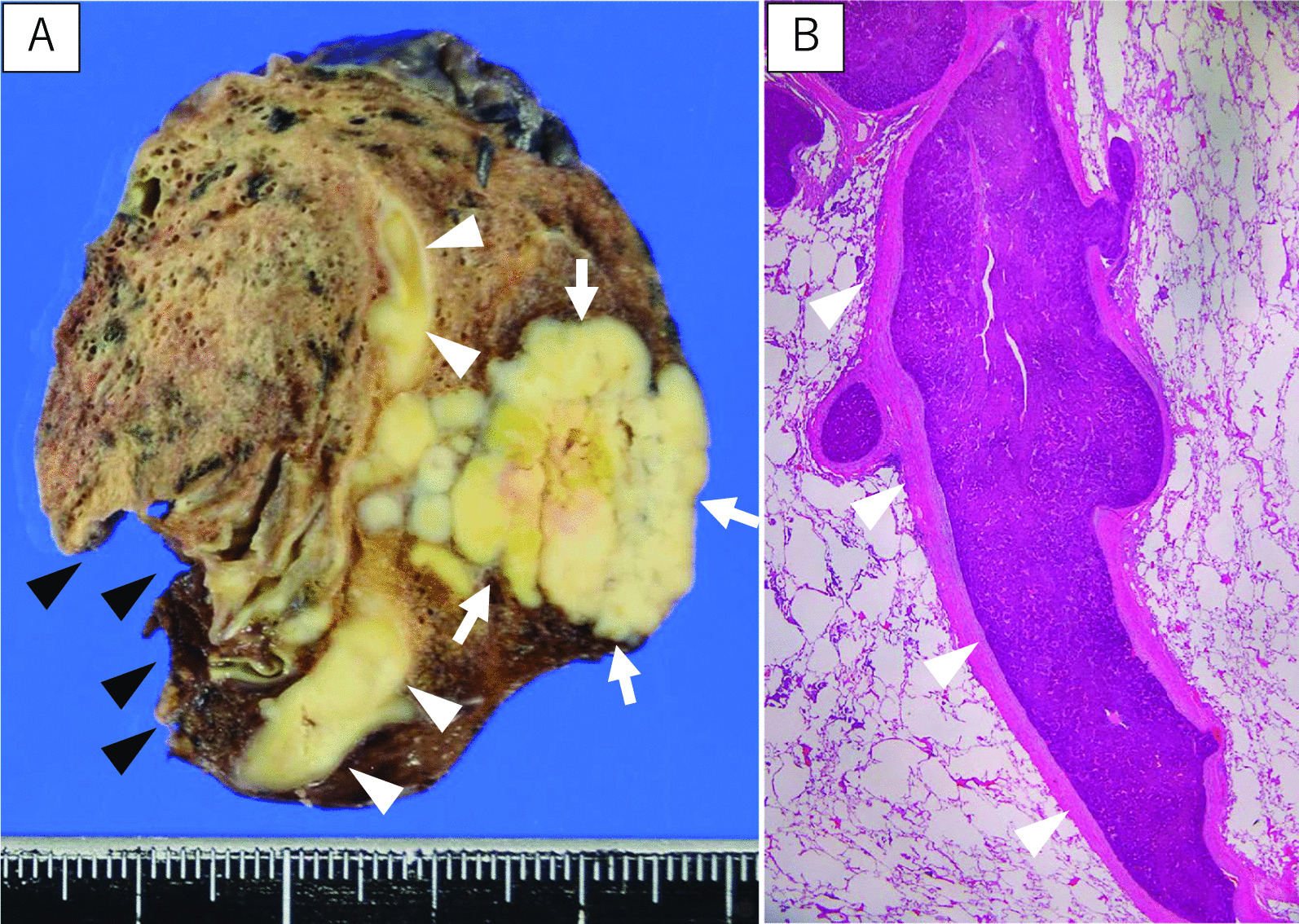
Resected specimen and micropathological findings. **A** Macroscopic findings of the resected specimen showing that the tumor invaded the pulmonary vein (white arrowhead). Cut end of the lung parenchyma (black arrowhead) is appropriately distant from the tumor (white arrow); **B** Micropathological examination revealing metastatic moderately differentiated hepatocellular carcinoma with tumor thrombus invasions in many pulmonary veins (white arrowhead)

A 77-year-old man who was hepatitis B virus carrier underwent laparoscopic lateral segment hepatectomy for HCC eight years ago. The pathological findings revealed no vessel invasion. He visited the hospital regularly for follow-ups. Protein induced by vitamin K absence or antagonist-II (PIVKA-II) remained elevated from two years ago, but imaging tests for the liver revealed no metastasis. Contrast-enhanced chest computed tomography (CT) showed a 27 mm nodule with mediastinal pleural invasion in the right apical segment (S1) (Fig. 1). The patient was pathologically diagnosed with a metastatic lung tumor from HCC via transbronchoscopic biopsy. We planned to perform right S1 segmentectomy because the resection margin was expected to be suboptimal by wedge resection. Before surgery, contrast-enhanced CT in the pulmonary vessel phase for three-dimensional (3D) reconstruction showed that the tumor extended into the adjusting peripheral pulmonary vein, and we diagnosed tumor thrombus invasion in V1a (Fig. 2). An additional movie file shows this in more detail [see Additional file 1]. The surgery was conducted under 3-port video-assisted thoracic surgery. The tumor had invaded the mediastinal pleura, so it was resected. First, the right superior pulmonary vein was exposed and the central part of V1 was ligated and cut to prevent tumor thrombus dispersal (Fig. 3). Tumor thrombus in V1a was not recognized before cut because the procedure for recognition of the extent of the tumor thrombus by palpation was risk for dispersal. Subsequently, A1 and B1 were cut respectively using a mechanical stapler. The intersegmental plane was identified by an inflation-deflation line after air inflation to the right lung, and subsequently cut with mechanical staplers. Finally, an intraoperative pathological examination of the frozen section demonstrated a malignant negative margin in V1. An additional movie file shows this in more detail [see Additional file 2]. The operative time was 185 min, and the operative blood loss was minimal. A prolonged postoperative pulmonary air leakage was resolved by the conservative treatment. The patient was discharged on postoperative day 14. Pathological examination revealed moderately-differentiated metastatic HCC with tumor thrombus invasion in many pulmonary veins, that was present contiguously with the tumor and contained cancer cells (Fig. 4). The molecular pattern resulted as follows; CK8/18, AMACR and HepPar1 were positive, and alpha-fetoprotein and Glyican-3 were negative. The features of this tumor were homologous to HCC in 8 years ago. Postoperatively, PIVKA-II returned to normal ranges and no additional treatments were performed. He has not shown the recurrence of HCC for 13 months.

## Discussion

As lung cancer has a high tendency to metastasize lymphogenously, it is necessary to perform lobectomy with systematic lymph node dissection. In contrast, because most metastatic lung tumors are developed by hematogenous metastasis, there is no need for systematic lymph node dissection, and the operative method only aims to resect the tumor. It has been reported that an insufficient resection margin is related to margin relapse; therefore, it is important to have a sufficient resection margin [5]. For lung cancer, the optimal resection margin was reported as a margin distance greater than 20 mm or the maximum tumor diameter [6]. For metastatic lung tumors from colorectal cancer, the optimal resection margin was reported to be a margin distance greater than 10 mm [7]. Wedge resection is often performed for metastatic lung tumors; however, segmentectomy or lobectomy is sometimes performed when the resection margin is expected to be suboptimal by wedge resection and the tumor is localized in the central lung field. In our case, we decided to perform segmentectomy for the optimal resection margin before diagnosis of the tumor thrombus in V1.

Advanced lung cancer sometimes invades in the left atrium via the pulmonary vein [8, 9]. In metastatic lung tumors, there have been some reports of tumor thrombus in the pulmonary vein. Most of them were metastatic lung tumors from HCC, renal cell carcinoma (RCC), and sarcoma [4, 10–13]. These malignant tumors have been reported to develop a tumor thrombus in the great vessels, such as the inferior vena cava, in primary lesions [3]. Most of metastatic lung tumors with a tumor thrombus were greater than 5 cm and invaded in the main pulmonary vein and the left atrium. Metastatic lung tumor < 3 cm in size, with a tumor thrombus only in the peripheral pulmonary vein, as in our case, is very rare.

Lung tumors with a tumor thrombus in the pulmonary vein have the potential to cause embolism in the systemic circulation as cerebral infarction by disengaging the tumor thrombus [14, 15]. It has been reported that lung tumors invading the pulmonary vein were detected after the development of transient ischemic attacks and cerebral infarction [16]. Additionally, embolism in the systemic circulation can occur during or after surgery [17]. If a tumor thrombus in the pulmonary vein can not be diagnosed preoperatively, an operative procedure such as resection of the pulmonary parenchyma and shifting of the lung without ligation of the pulmonary vein may cause embolism in the systemic circulation. To avoid such a situation, a preoperative diagnosis of a tumor thrombus and ligation of the pulmonary vein at the beginning of the surgery is necessary.

**Fig. 5:**
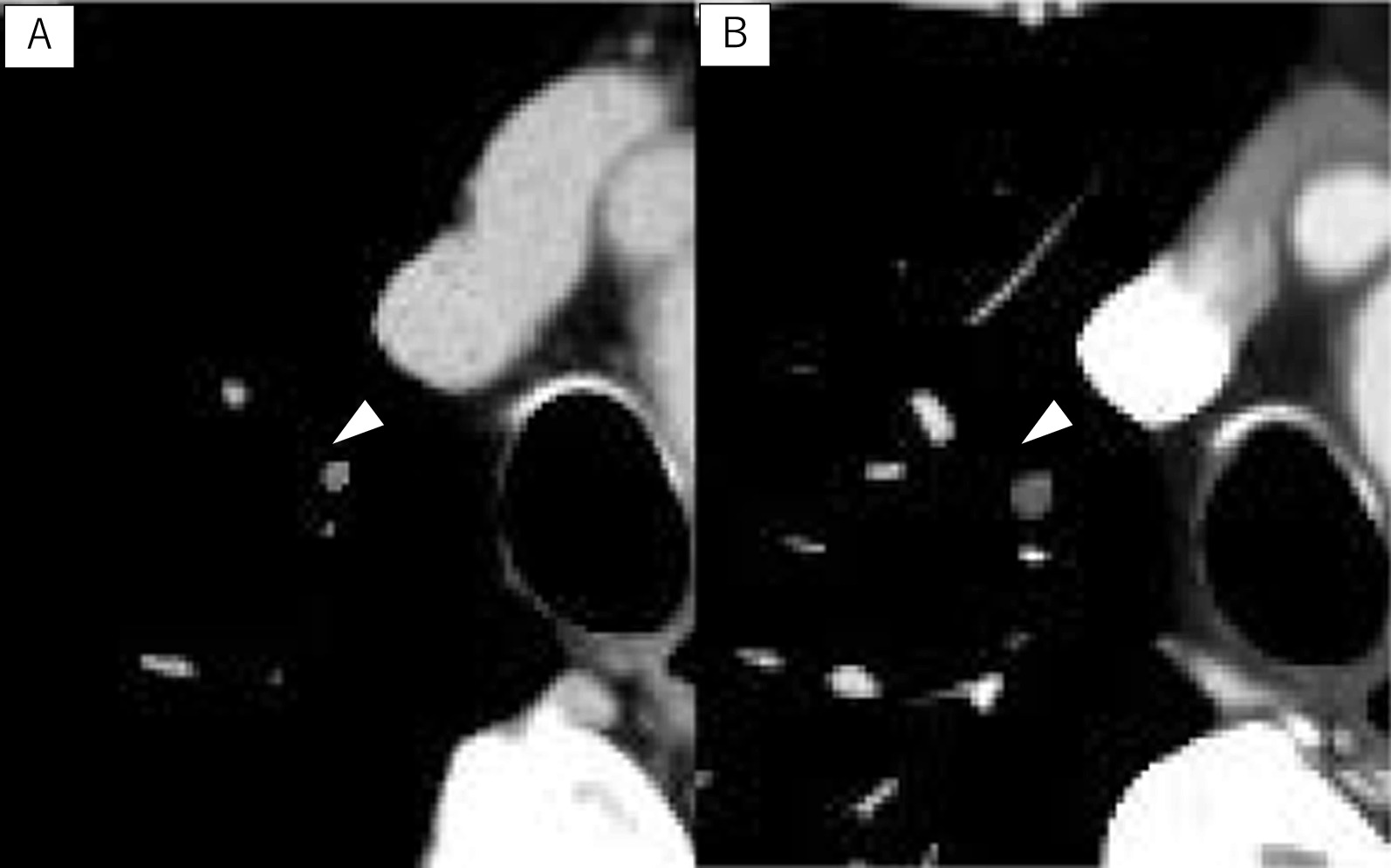
Comparison between standard contrast-enhance CT and contrast-enhanced CT in the pulmonary vessel phase. **A** Standard contrast-enhanced CT showing a tumor thrombus in the pulmonary vein (white arrowhead) could not be detected; **B** contrast-enhanced CT in the pulmonary vessel phase showing a tumor thrombus in the pulmonary vein (white arrowhead) was evident because of the difference from the other contrast-filled vessels

It is recommended to perform contrast-enhanced CT in the pulmonary vessel phase for 3D reconstruction to understand the anatomical features of the pulmonary vessels and bronchi before performing segmentectomy and lobectomy [18]. Nakahashi et al. reported that pulmonary venous tumor thrombus could be diagnosed by contrast-enhanced CT in the pulmonary venous phase; however, this was not by standard contrast-enhanced CT [4]. In our case, the tumor thrombus in V1a could not be diagnosed using the standard contrast-enhanced CT (Fig. 5). Without a preoperative diagnosis, an operative procedure without first ligation of V1 might cause embolism by a tumor thrombus during the operation. Malignant tumors tend to invade the vessels and develop a tumor thrombus, such as HCC, RCC, and sarcoma. It might be necessary to perform preoperative contrast-enhanced CT in the pulmonary vessel phase to check for a tumor thrombus when surgery for metastatic lung tumor is performed.

## Conclusions

We report a case of a metastatic lung tumor from HCC with tumor thrombus invasion in the pulmonary vein that was diagnosed preoperatively and underwent complete resection by segmentectomy. Without a preoperative diagnosis of a tumor thrombus, it may cause embolism in the systemic circulation during an operation. As malignant tumors tend to develop a tumor thrombus in the primary tumor, it might be necessary to perform contrast-enhanced CT in the pulmonary vessel phase to check for a tumor thrombus before the operation for metastatic lung tumors.

## Supplementary Information

**Additional file 1:** Contrast-enhanced CT in the pulmonary vessel phase for three-dimensional reconstruction.

**Additional file 2:** Intraoperative findings by surgical video.

## Data Availability

Please contact the author for data requests.

## Abbreviations

HCC: Hepatocellular carcinoma
PIVKA-II: Protein induced by vitamin K absence or antagonist-II
S1: Apical segment
3D: Three-dimensional
RCC: Renal cell carcinoma
